# Letter from the Editor in Chief

**DOI:** 10.19102/icrm.2020.110401

**Published:** 2020-04-15

**Authors:** Moussa Mansour


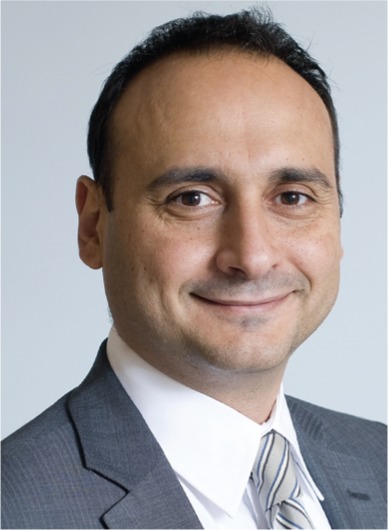


Dear Readers,

I would like to begin my letter by extending sincere gratitude and well wishes to health care professionals around the world who are valiantly fighting the COVID-19 pandemic on the frontlines during the current unprecedented health care crisis. We stand united with you.

“Never in the field of human conflict was so much owed by so many to so few.” This is a famous line from a speech by British Prime Minister Winston Churchill on August 20, 1940 during the outset of World War II, when referring to the heroic ongoing efforts of Royal Air Force crews who were, at the time, fighting the Battle of Britain, the pivotal air battle with the German Luftwaffe. This quote briefly encapsulates the sentiments that we all have for the overburdened and understaffed health care professionals who are risking their lives to save others during the ongoing global pandemic.

The impact of COVID-19 has sparked far-ranging impacts on the global landscape of the health care field, forcing governments, hospitals, businesses, educational institutions, and citizens to enact unprecedented measures to combat the spread of the contagion and aid in the restoration of economies.

As a result, government agencies and hospitals have issued guidance to postpone elective procedures until the transmission of the contagion has abated. The field of cardiac electrophysiology has been severely affected by this important and necessary policy. Large numbers of catheter ablation procedures and device implants have been canceled. Performing the backlog of procedures in the future will require significant effort and countless night and weekend shifts. This step of ceasing such procedures in the meantime, however, might be our main contribution in this battle, because it helps direct all hospital resources toward fighting the COVID-19 outbreak. In addition to postponing elective procedures, many electrophysiologists are volunteering to perform general medicine tasks and provide care for sick patients battling the virus despite their lack of expertise in those fields.

Despite the severity of this crisis and the seemingly endless nature of it, I believe that bright days are ahead of us. As health care professionals, our resilience, determination, innate nature to save lives, and creativity, will guide us to reach the light at the end of the tunnel.

Best wishes for a safe outcome to all of you.

Sincerely,


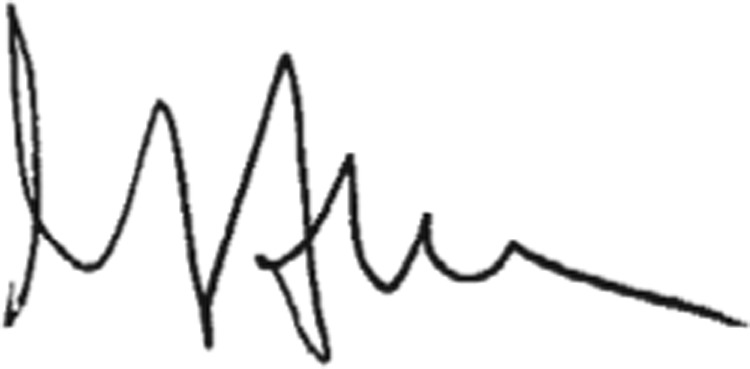


Moussa Mansour, md, fhrs, facc

Editor in Chief

The Journal of Innovations in Cardiac Rhythm Management

MMansour@InnovationsInCRM.com

Director, Atrial Fibrillation Program

Jeremy Ruskin and Dan Starks Endowed Chair in Cardiology

Massachusetts General Hospital

Boston, MA 02114

